# Supplementary material for: A framework for near-real time monitoring of diversity patterns based on indirect remote sensing, with an application in the Brazilian Atlantic rainforest
Source: PeerJ. 2022 Jun 29;10:e13534. doi: 10.7717/peerj.13534 (PMC9250313; doi:10.7717/peerj.13534)
Supplement: Supplemental Information 1 [file peerj-10-13534-s001.docx]

**Supplemental Material**

**Tables**

Table S1. Results and tuned parameters for individual machine learning models: Random Forests (RF, rf from Liaw & Wiener, 2002), Neural Network (NN, nnet from Venables & Ripley, 2002), Support Vector Machines (SVM, svmRadial from Karatzoglou et al., 2004), and Generalized Linear Models (GLM) used in the final ensemble modelling.

| Biological Group | Diversity metric | Model | R2 | Final tuned parameters |
| --- | --- | --- | --- | --- |
| *Bertolonia* | Species richness | RF | 0.29 | mtry=2 |
|  |  | NN | 0.13 | size = 3; decay = 0 |
|  |  | SVM | 0.14 | C=0.5 (sigma constant= 0.12) |
|  |  | GLM | 0.17 |  |
|  | Phylogenetic diversity | RF | 0.28 | mtry = 2 |
|  |  | NN | 0.16 | size = 1; decay = 1e-4 |
|  |  | SVM | 0.24 | C=1 (sigma constant= 0.17) |
|  |  | GLM | 0.05 |  |
| *Fridericia & allies* | Species richness | RF | 0.7 | mtry=6 |
|  |  | NN | 0.67 | size =5 decay = 0 |
|  |  | SVM | 0.67 | C=0.24 (sigma constant 0.11) |
|  |  | GLM | 0.54 |  |
|  | Phylogenetic diversity | RF | 0.62 | mtry=2 |
|  |  | NN | 0.56 | size=5 decay = 1e-04 |
|  |  | SVM | 0.6 | C=0.25 (sigma constant = 0.11) |
|  |  | GLM | 0.44 |  |
| Bromelioideae | Species richness | RF | 0.27 | mtry=2 |
|  |  | NN | 0.29 | size=5, decay = 0 |
|  |  | SVM | 0.13 | C=1 (sigma constant= 0.13) |
|  |  | GLM | 0.19 |  |
|  | Phylogenetic diversity | RF | 0.27 | mtry=2 |
|  |  | NN | 0.23 | size =5 decay = 0.1 |
|  |  | SVM | 0.25 | C=1 (sigma constant= 0.12) |
|  |  | GLM | 0.18 |  |
| Ithomiini | Species richness | RF | 0.57 | mtry = 2 |
|  |  | NN | 0.39 | size = 3 decay = 0 |
|  |  | SVM | 0.6 | C= 0.5 (sigma constant = 0.12) |
|  |  | GLM | 0.36 |  |
|  | Phylogenetic diversity | RF | 0.57 | mtry = 2 |
|  |  | NN | 0.51 | size = 5 decay = 0.1 |
|  |  | SVM | 0.56 | C= 0.0.25 (sigma constant = 0.12) |
|  |  | GLM | 0.38 |  |
| Cambessedesieae | Species richness | RF | 0.34 | mtry=2 |
|  |  | NN | 0.33 | size = 3, decay = 1e-04 |
|  |  | SVM | 0.32 | C = 0.25 (sigma constant = 0.11) |
|  |  | GLM | 0.21 |  |
|  | Phylogenetic diversity | RF | 0.13 | mtry = 2 |
|  |  | NN | 0.18 | size = 5 decay = 1e-04 |
|  |  | SVM | 0.16 | C = 1 (sigma constant = 0.12) |
|  |  | GLM | 0.15 |  |
| *Boana* | Species richness | RF | 0.44 | mtry = 2 |
|  |  | NN | 0.39 | size = 5, decay = 0.1 |
|  |  | SVM | 0.33 | C = 0.5 (sigma constant = 0.13) |
|  |  | GLM | 0.38 |  |
|  | Phylogenetic diversity | RF | 0.48 | mtry = 2 |
|  |  | NN | 0.41 | size = 5 decay = 1e-04 |
|  |  | SVM | 0.42 | C = 0.25 (sigma constant = 0.12) |
|  |  | GLM | 0.35 |  |
| Miconieae | Species richness | RF | 0.52 | mtry = 6 |
|  |  | NN | 0.66 | size = 1; decay = 1e-4 |
|  |  | SVM | 0.6 | C = 0.25 (sigma constant = 0.11) |
|  |  | GLM | 0.6 |  |
|  | Phylogenetic diversity | RF | 0.56 | mtry = 2 |
|  |  | NN | 0.53 | size = 5, decay = 0 |
|  |  | SVM | 0.59 | C = 0.25 (sigma constant = 0.12) |
|  |  | GLM | 0.62 |  |
| *Proceratophrys* | Species richness | RF | 0.21 | mtry = 2 |
|  |  | NN | 0.11 | size = 5, decay = 0.1 |
|  |  | SVM | 0.17 | C= 1 (sigma constant = 0.12) |
|  |  | GLM | 0.09 |  |
|  | Phylogenetic diversity | RF | 0.13 | mtry = 2 |
|  |  | NN | 0.058 | size = 1, decay = 0 |
|  |  | SVM | 0.12 | C = 0.25 (sigma constant = 0.12) |
|  |  | GLM | 0.07 |  |
| Thraupinae | Species richness | RF | 0.58 | mtry = 2 |
|  |  | NN | 0.6 | size = 1, decay = 0.1 |
|  |  | SVM | 0.63 | C = 0.5 (sigma constant = 0.11) |
|  |  | GLM | 0.61 |  |
|  | Phylogenetic diversity | RF | 0.56 | mtry = 2 |
|  |  | NN | 0.4 | size = 5, decay = 0 |
|  |  | SVM | 0.61 | C = 0.5 (sigma constant = 0.12) |
|  |  | GLM | 0.45 |  |

Table S2. Percent contribution of each variable to predictive models. Most important variable for each group is in bold. Variables are one vegetation index (NDVI), temperature related (bio 2, bio 3, bio 4, bio 8, and bio 9) and precipitation related (bio 13, bio 14, bio 18 and bio 19). Results are shown for each diversity measure and each group.

|  | Contribution to the model (%) | | | | | | | | | |
| --- | --- | --- | --- | --- | --- | --- | --- | --- | --- | --- |
|  | Species richness | | | | | Phylogenetic diversity | | | | |
| Variable | Miconieae | Fridericia & allies | Thraupinae | Ithomiini | *Boana* | Miconieae | Fridericia & allies | Thraupinae | Ithomiini | *Boana* |
| NDVI | 2.01 | 9.84 | 4.03 | 14.33 | 3.44 | 2.55 | 9.01 | 2.36 | 14.01 | 4.83 |
| bio2 | 8.19 | 8.72 | 7.13 | 8.53 | 2.83 | 5.42 | 11.84 | 3.15 | 7.78 | 2.77 |
| bio3 | 8.99 | 7.92 | 8.77 | 8.29 | 15.47 | 10.11 | 11.18 | 7.21 | 5.74 | 18.76 |
| bio4 | 14.70 | 5.98 | 9.37 | 6.16 | 7.30 | 11.46 | 6.83 | 5.17 | 5.15 | 5.43 |
| bio8 | 7.27 | 6.52 | 4.70 | 11.18 | 7.46 | 11.87 | 5.44 | 4.62 | 14.15 | 4.9 |
| bio9 | 14.17 | 5.36 | 13.82 | 4.08 | 5.18 | 8.08 | 6.22 | 16.20 | 7.51 | 8.41 |
| bio13 | 10.42 | **17.86** | 13.09 | **20.09** | 6.96 | 12.14 | **14.63** | **21.39** | **16.88** | 7.15 |
| bio14 | 2.09 | 10.86 | 7.46 | 7.11 | **21.83** | 6.02 | 13.92 | 8.86 | 8.74 | 16.7 |
| bio18 | **25.31** | 9.74 | **18.27** | 9.78 | 15.23 | **24.60** | 10.26 | 20.34 | 12.33 | **22.05** |
| bio19 | 6.86 | 17.18 | 13.38 | 10.46 | 14.29 | 7.75 | 10.68 | 10.71 | 7.7 | 9 |

**Figures**


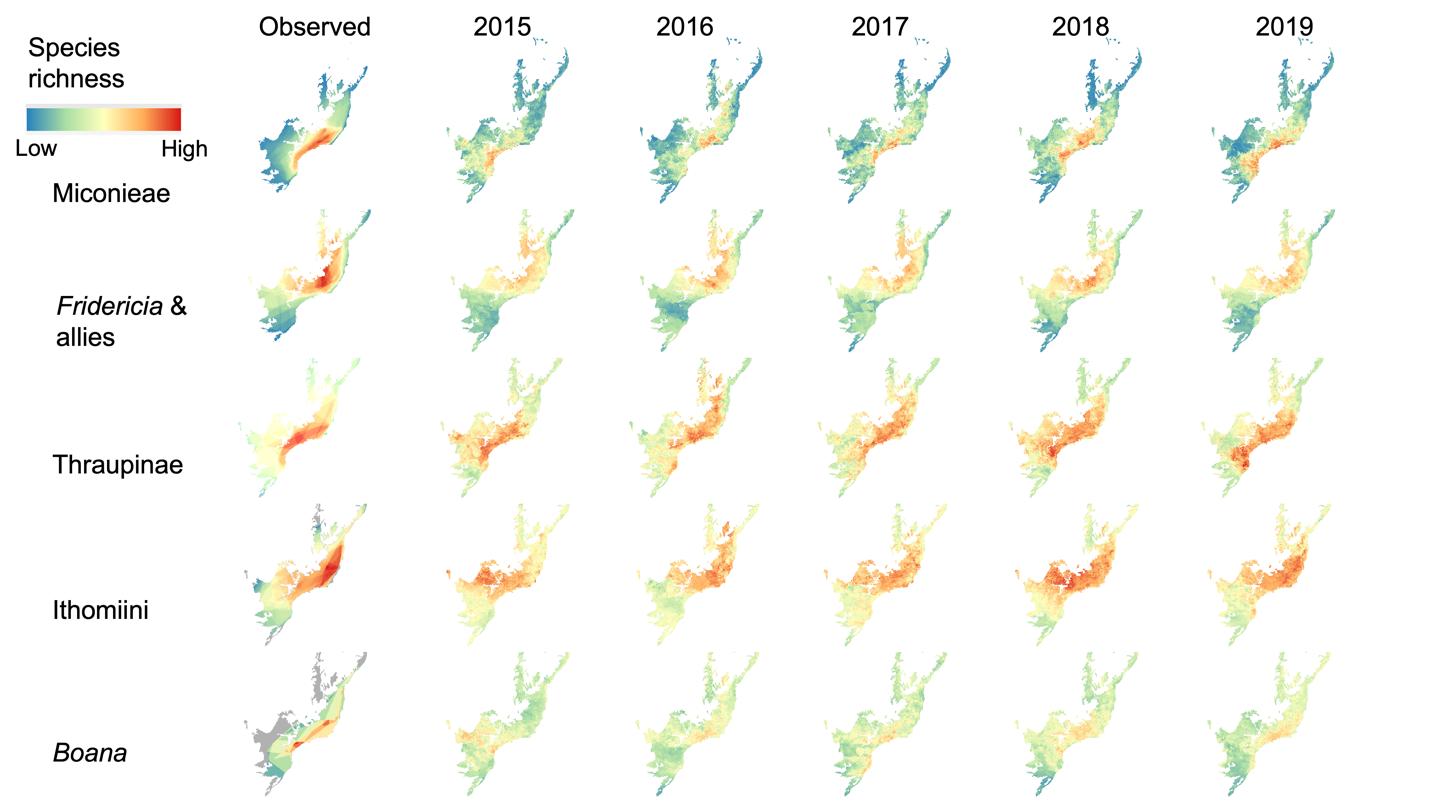


Figure S1. Models of temporal change in species richness between the years of 2015 and 2019, for the five groups with highest model predictive ability (R2 >0.5): From top to bottom: Miconieae, the Fridericia & allies, the Thraupinae birds, the Ithomiini butterflies, and the Boana frogs. A species richness map is provided, based on data pooled from the years prior to model training (pre-2015; “observed”), along with maps of the predicted species richness at each year. Warmer colors represent higher species richness, colder colors depict lower species richness. Maps are scaled similarly in each group (ranging from the minimum to maximum value observed in all maps).


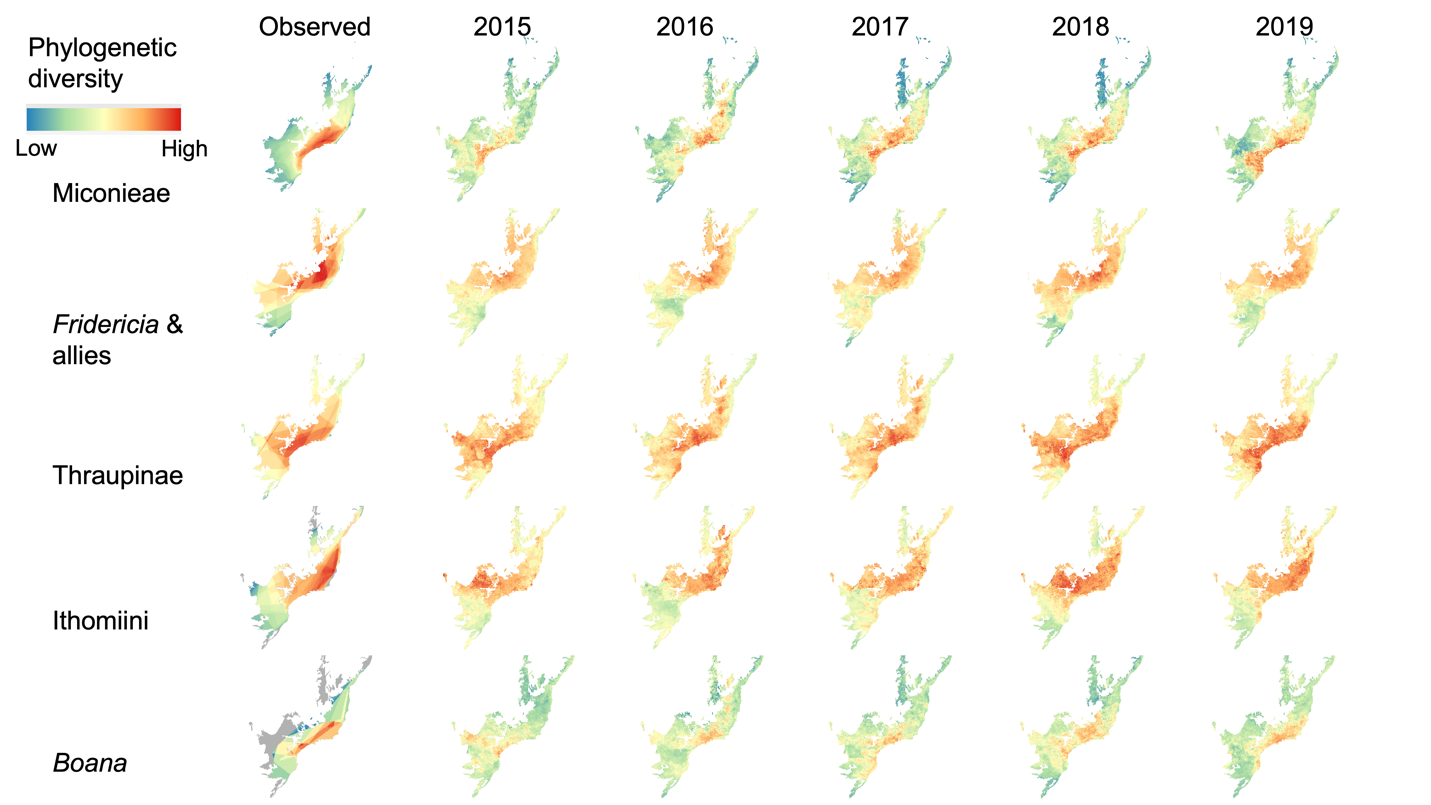


Figure S2. Models of temporal change in phylogenetic diversity between the years of 2015 and 2019, for the three groups showing highest model predictive ability (R2 >0.5): From top to bottom: Miconieae, the Fridericia & allies, the Thraupinae birds, the Ithomiini butterflies, and the Boana frogs. A phylogenetic diversity map is provided, based on data pooled from the years prior to model training (pre-2015; “observed”), along with maps of the predicted species richness at each year. Warmer colors represent higher species richness, colder colors depict lower species richness. Maps are scaled similarly in each group (ranging from the minimum to maximum value observed in all maps).


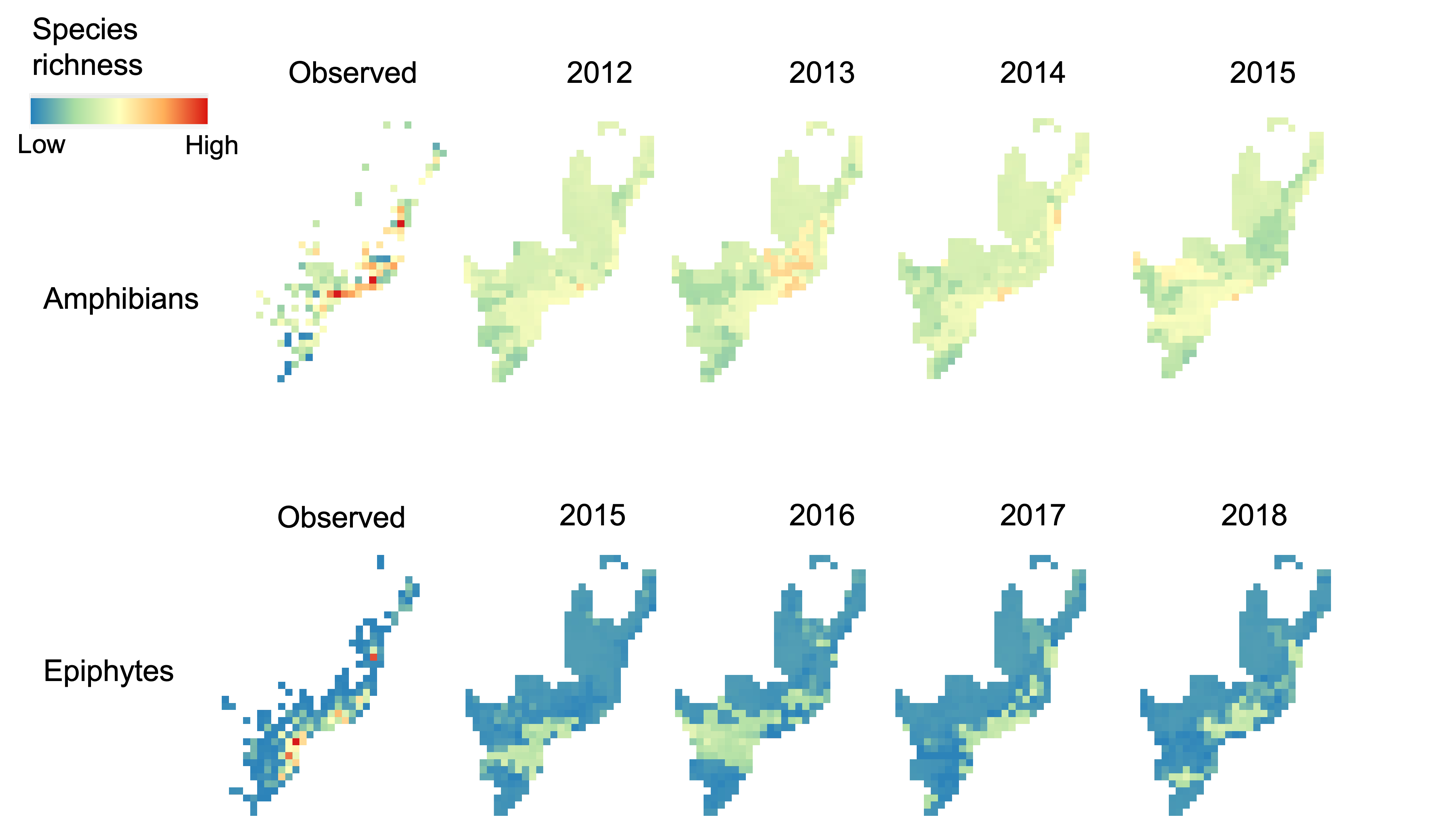


Figure S3. Models of temporal change in species richness built from the ATLANTIC-amphibians dataset (top, projected for the years of 2012 to 2015) and from the ATLANTIC-epiphytes dataset (bottom, projected for the years 2015 to 2018). A species richness map is provided, based on data pooled from the years prior to model training (pre-2012 in amphibians, pre-2015 in epiphytes; “observed”), along with maps of the predicted species richness at each year. Warmer colors represent higher species richness, colder colors depict lower species richness. Maps are scaled similarly in each group (ranging from the minimum to maximum value observed in all maps).

Figure S4. Variation in predictor variables in the years used for model projection. From left to right: 2015, 2016,2017,2018 and 2019. Variation is shown for mean annual temperature (Bio 1; top), Annual precipitation (Bio 12; middle) and Median NDVI (bottom).
